# Biological Age is a predictor of mortality in Ischemic Stroke

**DOI:** 10.1038/s41598-018-22579-0

**Published:** 2018-03-07

**Authors:** Carolina Soriano-Tárraga, Eva Giralt-Steinhauer, Marina Mola-Caminal, Angel Ois, Ana Rodríguez-Campello, Elisa Cuadrado-Godia, Israel Fernández-Cadenas, Natalia Cullell, Jaume Roquer, Jordi Jiménez-Conde

**Affiliations:** 1 Department of Neurology, Hospital del Mar; Neurovascular Research Group, IMIM (Institut Hospital del Mar d’Investigacions Mèdiques); Universitat Autònoma de Barcelona/DCEXS-Universitat Pompeu Fabra, Barcelona, Spain; 2 Stroke Pharmacogenomics and Genetics, Fundació Docència i Recerca, MutuaTerrassa, Hospital Mútua de Terrassa, Terrassa, Spain

## Abstract

Age and stroke severity are the main mortality predictors after ischemic stroke. However, chronological age and biological age are not exactly concordant. Age-related changes in DNA methylation in multiple CpG sites across the genome can be used to estimate biological age, which is influenced by lifestyle, environmental factors, and genetic variation. We analyzed the impact of biological age on 3-month mortality in ischemic stroke. We assessed 594 patients with acute ischemic stroke in a cohort from Hospital del Mar (Barcelona) and validated the results in an independent cohort. Demographic and clinical data, including chronological age, vascular risk factors, initial stroke severity (NIHSS score), recanalization treatment, and previous modified Rankin scale were registered. Biological age was estimated with an algorithm based on DNA methylation in 71 CpGs. Biological age was predictive of 3-month mortality (p = 0.041; OR = 1.05, 95% CI 1.00–1.10), independently of NIHSS score, chronological age, TOAST, vascular risk factors, and blood cell composition. Stratified by TOAST classification, biological age was associated with mortality only in large-artery atherosclerosis etiology (p = 0.004; OR = 1.14, 95% CI 1.04–1.25). As estimated by DNA methylation, biological age is an independent predictor of 3-month mortality in ischemic stroke regardless of chronological age, NIHSS, previous modified Rankin scale, and vascular risk factors.

## Introduction

After an ischemic stroke (IS), determining the individual patient’s risk of mortality at hospital admission has great clinical relevance and provides critical information for patients and their families^1–4^. DNA methylation (DNAm) is an epigenetic mechanism regulating higher-order DNA structure and gene expression. It is a heritable but also reversible addition of a methyl group to the 5-carbon position of cytosine in a cytosine-phosphate-guanine (CpG) context, associated with gene silencing^5^. DNAm varies across the lifespan and its levels are influenced by lifestyle and environmental factors, as well as by genetic variation^6–8^.

Age-related changes in DNAm are well documented, and two recent studies used methylation measured in multiple CpGs across the genome to predict *chronological age* (c-Age) in humans^9,10^. Using the Illumina BeadChip, Hannum *et al*.^9^ created an age predictor based on whole blood DNA. The difference between chronological and methylation-predicted age, defined as average age acceleration, can be used to determine whether the DNAm age, also called *epigenetic age* or *biological age* (b-Age), is consistently higher or lower than expected. We previously reported that IS patients are biologically older (a mean of 2.5 years), than controls of the same c-Age and that b-Age is a better predictor of 3-month outcome^11,12^.

A recent meta-analysis of 13 cohorts found that epigenetic age acceleration predicts all-cause mortality, independent of c-Age and even after adjusting for traditional risk factors^13^. Nevertheless, the prevalent disease status of the participants evaluated did not include stroke, one of the leading causes of mortality^14^. The aim of our study was to assess the b-Age contribution to IS mortality at 3 months and to stratify the analysis by stroke subtypes.

## Results

A total of 594 Caucasian patients with IS were included in the discovery analysis. Detailed descriptions of the discovery and replication cohorts are summarized in Table 1. In the discovery and replication cohorts, IS mortality at 3 months was 15.8% and 17.6%, respectively. The b-Age estimates had a strong positive correlation with c-Age (r = 0.81).

**Table 1:** Baseline characteristics of ischemic stroke patients in the discovery and replication cohorts.

Characteristics	Discovery N = 594	Replication N = 85	p-value
Age*	77 (68–83)	74 (66–80)	0.153
Sex, female, n (%)	267/594 (44.9)	25/85 (29.4)	0.007
Dyslipidemia, n (%)	276/594 (46.5)	35/83 (42.2)	0.462
Hypertension, n (%)	430/594 (72.4)	53 (62.4)	0.056
Diabetes mellitus, n (%)	248/594 (41.8)	20/85 (23.5)	0.001
Coronary heart disease, n (%)	90/592 (15.2)	15/84 (17.9)	0.530
Atrial fibrillation, n (%)	220/594 (37.0)	20/83 (24.1)	0.021
Smoking habit, n (%):			<0.001
Current/ Former (<5 years)	279/592 (47.1)	20/83 (24.1)	
Never smokers	313/592 (52.9)	63/83 (75.9)	
Ischemic stroke etiology, n (%)			<0.001
Large-artery atherosclerosis	153/594 (25.8)	35/84 (41.7)	
Small-vessel disease	199/594 (33.5)	8/84 (9.5)	
Cardioembolism	242/594 (40.7)	19/84 (22.6)	
Undetermined	—	22/84 (26.2)	
NIHSS score*	5 (3–12)	9 (3–17)	0.006
Recanalization treatment, n (%)	97/594 (16.3)	42/82 (51.2)	<0.001
Previous mRS*			<0.001
0	375/594 (63.1)		
1	73/594 (12.3)	85/85 (100)	
2	62/594 (10.4)		
3	66/594 (11.1)	—	
4	16/594 (2.7)	—	
5	2/594 (0.3)	—	
3-month mortality, n (%)	94/594 (15.8)	15/85 (17.6)	0.669

*Median (Interquartile range).

NIHSS, National Institutes of Health Stroke Scale; mRS, modified Rankin Scale.

Bivariate analysis for IS mortality at 3 months is summarized in Table 2. Significant variables were c-Age, b-Age, sex, previous modified Rankin Scale (p-mRS), initial National Institutes of Health Stroke Scale (NIHSS), recanalization treatment, dyslipidemia, coronary artery disease, atrial fibrillation, smoking habit, and IS etiology (TOAST,trial of ORG 10172 in acute stroke treatment classification). No statistical differences were observed for hypertension and diabetes mellitus.

**Table 2:** Mortality at 3 months after IS. Bivariate analysis.

	Alive (N = 500)	Deceased (N = 94)	p-value
Chronological age (years)*	76 (66–82)	83 (75–87)	<0.001
Biological age (years)*	73.1 (66.7–78.6)	80.1 (73.9–84.3)	<0.001
Sex (female), n (%)	209 (41.8)	58 (61.7)	<0.001
Previous mRS*	0 (0–1)	1 (0–3)	<0.001
NIHSS*	4 (2–8)	17 (12–20)	<0.001
Recanalization treatment, n (%)	75 (15.0)	22 (23.4)	0.043
Dyslipidemia, n (%)	244 (48.8)	32 (34.0)	0.008
Hypertension, n (%)	358 (71.6)	72 (76.6)	0.320
Diabetes mellitus, n (%)	208 (41.6)	40 (42.6)	0.863
Coronary heart disease, n (%)	69 (13.9)	21 (22.3)	0.036
Atrial fibrillation, n (%)	148 (29.6)	72 (76.6)	<0.001
Smoking habit, n (%)	253 (50.7)	26 (28.2)	<0.001
Ischemic stroke etiology, n (%)			<0.001
Large-artery atherosclerosis	134 (26.8)	19 (20.2)	
Small-vessel disease	198 (39.6)	1 (1.1)	
Cardioembolism	168 (33.6)	74 (78.7)	

*Median (Interquartile range).

^†^Mean (Standard deviation).

**Figure 1:**
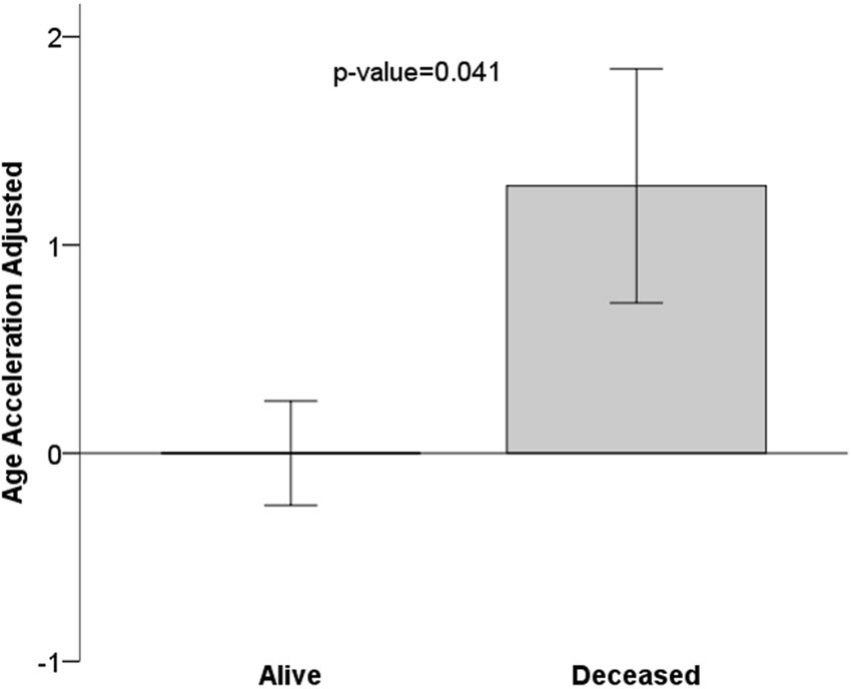
Mean age acceleration relates to mortality, p‐value of Student T-test. Age acceleration is the linear regression of biological age adjusted by chronological age, sex, stroke severity, TOAST and p-mRs. By definition, the mean age of participants alive at 3 months is zero. Each bar plot reports 1 standard error. TOAST, trial of ORG 10172 in acute stroke treatment; p-mRs, previous modified Rankin scale.

Logistic multivariate regression analysis of mortality at 3 months after IS (Table 3) consisted of three models. Model 1 was fully adjusted, including all 11 variables from the bivariate analysis (above), and the Nagelkerke-adjusted R-squared had an explanatory power of 0.501. There was an independent association between b-Age and mortality [p = 0.037; odds ratio (OR) = 1.06 (95% CI: 1.00–1.11)], nullifying c-Age (p = 0.644). For model 2, a stepwise regression model, the explanatory power of R-squared was 0.493. The most efficient model included b-Age, previous mRS, initial NIHSS, and IS etiology. In this model, b-Age was statistically associated with mortality [p = 0.001; OR = 1.06 (95% CI: 1.02–1.10)], independently of initial NIHSS [p < 0.001; OR = 1.18 (95% CI 1.13–1.24)], previous functional condition (mRS) [p = 0.003; OR = 1.37 (95% CI: 1.11–1.68)], and TOAST (p = 0.015), (Fig. 1). Small-vessel disease (SVD) etiology remained as a protective factor against mortality [p = 0.020; OR = 0.09 (95% CI: 0.01–0.68], taking as reference large-artery atherosclerosis (LAA). Given that age-related changes in blood cell composition are well documented, model 3 was further adjusted by blood cell proportions; b-Age remained statistically significant.

**Table 3:** Logistic multivariate regression models of mortality at 3 months after IS. Model 1 is the fully adjusted model, adjusted by clinical covariates and biological age. In model 2, stepwise logistic regression selected the most efficient model using the variables included in model 1. Model 3 is model 2 with results for all blood cell compositions.

	Model 1 (N = 590)		Model 2 (N = 594)		Model 3 (N = 594)	
	P- value	OR (95% CI) R^2^ = 0.501	P- value	OR (95% CI) R^2^ = 0.493	P- value	OR (95% CI) R^2^ = 0.541
Biological age	0.037	1.06 (1.00–1.11)	0.001	1.06 (1.02–1.10)	0.041	1.05 (1.00–1.10)
Chronological age	0.644	1.01 (0.96–1.06)	—	—	—	—
Sex, female	0.933	1.09 (0.97–2.08)	—	—	—	—
Previous mRS	0.011	1.33 (1.07–1.67)	0.003	1.37 (1.11–1.68)	0.006	1.37 (1.10–1.70)
NIHSS	<0.001	1.19 (1.13–1.25)	<0.001	1.18 (1.13–1.24)	<0.001	1.19 (1.13–1.25)
Recanalization treatment	0.610	0.83 (0.40–1.72)	—	—	—	—
Dyslipidemia	0.363	0.76 (0.41–1.38)	—	—	—	—
Atrial fibrillation	0.639	1.45 (0.31–6.91)	—	—	—	—
Coronary heart disease	0.202	1.62 (0.77–3.39)	—	—	—	—
Smoking habit	0.366	1.27 (0.76–2.11)	—	—	—	—
Ischemic stroke etiology:	0.064	—	0.015	—	0.013	
LAA	ref	—	ref	—	ref	
SVD	0.020	0.09 (0.01–0.68)	0.020	0.09 (0.01–0.68)	0.028	0.07 (0.01–0.75)
CE	0.922	1.08 (0.22–5.39)	0.229	1.49 (0.78–2.84)	0.120	1.74 (0.87–3.48)
Blood cell estimation:	—	—	—	—	—	—
Monocytes	—	—	—	—	0.054	—
NK	—	—	—	—	0.471	—
B cells	—	—	—	—	0.884	—
Granulocytes	—	—	—	—	0.261	—
CD4 T cells	—	—	—	—	0.263	—
CD8 T cells	—	—	—	—	0.285	—
naïve CD8 T cells	—	—	—	—	0.378	—
naïve CD4 T cells	—	—	—	—	0.472	—
CD8 + CD28‐CD45RA‐	—	—	—	—	0.551	—
Plasmablast	—	—	—	—	0.013	14.8 (1.75–125)

OR, odds ratio; CI, confidence interval; R^2^ adjusted-R squared (adjusted for the number of predictors in the model); NIHSS, National Institutes of Health Stroke Scale; mRS, modified Rankin Scale LAA, large-artery atherosclerosis; SVD, small vessel disease; CE, cardioembolism.

The results were successfully validated in a replication cohort (VH) with a 3-month mortality of 17.6% (N = 15). The b-Age estimates also had a strong positive correlation with c-Age (r = 0.77). Demographics characteristic of the VH cohort are summarized in Table 1 and Supplemental Table S1. Its limited sample size restricted the number of variables used to adjust the analysis. Model 1 was adjusted by b-Age, c-Age, sex, initial NIHSS, and recanalization treatment; R-squared was 0.441. Again, b-Age was associated with mortality [p = 0.045; OR = 1.14 (95% CI: 1.00–1.29)], nullifying c-Age (p = 0.424); initial NIHSS was the most significant variable [p = 0.013; OR 1.22 (95% CI: 1.04–1.43)]. Recanalization treatment and sex were not significant. At 3 months, b-Age was an independent predictor of mortality after an IS.

It is known that mortality is mostly associated with LAA and cardioembolic (CE) etiologies; single-vessel disease (SVD) is associated with less severe strokes (Table 3). Moreover, the mortality influence of c-Age may differ depending on stroke subtype. For this reason, we also stratified the discovery data by IS etiology. Bivariate analysis of IS mortality at 3 months, stratified by TOAST, is summarized in Supplemental Table S2.

**Figure 2:**
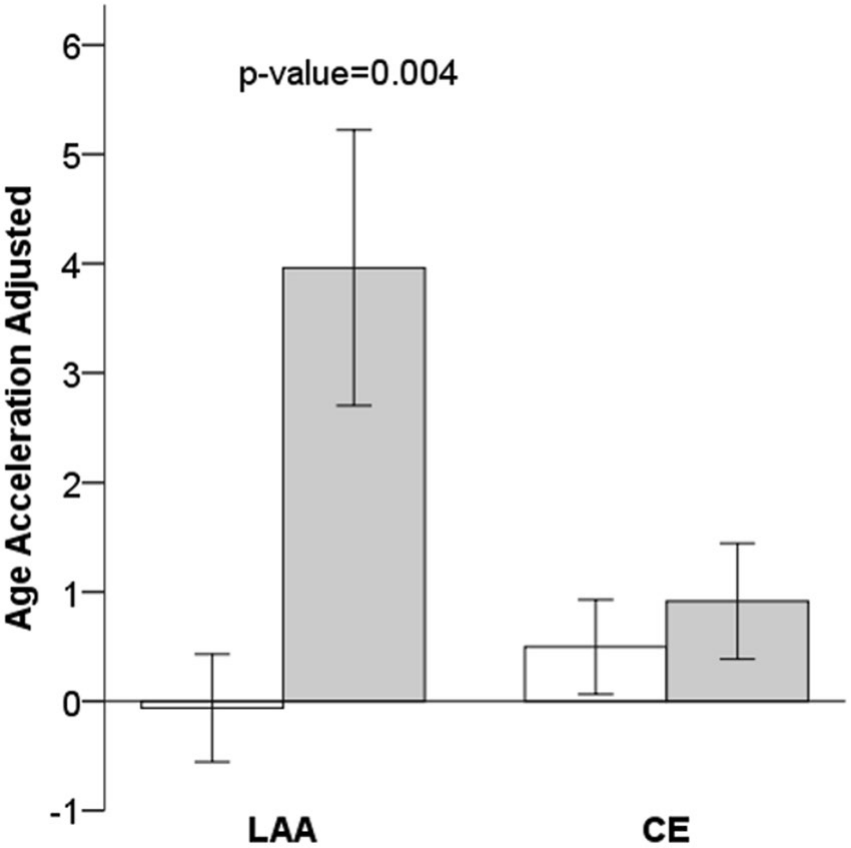
Mean age acceleration associated to mortality, stratified by stroke etiology: alive (white bars); deceased (grey bars); and p‐value of Student T-test. Age acceleration was adjusted by chronological age, sex, stroke severity, TOAST and p-mRs values. Each bar plot reports 1 standard error. LAA, large-artery atherosclerosis; CE, cardioembolic; TOAST, trial of ORG 10172 in acute stroke treatment; p-mRs, previous modified Rankin scale.

Multivariate logistic regression analysis of 3-month mortality stratified by TOAST is shown in Table 4. With only one SVD death in the sample, this etiology was not included. For LAA etiology (N = 153), model 1 is the fully adjusted model, including all variables that were statistically significant in the bivariate analysis; the explanatory power of R-squared was 0.373. There was an independent association between b-Age and mortality [p = 0.004; OR = 1.14 (95% CI: 1.04–1.25)], nullifying c-Age (p = 0.342). For model 2, the stepwise regression model, the explanatory power of R-squared was 0.281. The most efficient model included b-Age (p = 0.006; OR = 1.10 (95% CI: 1.03–1.18)] and initial NIHSS [p < 0.001; OR = 1.17 (95% CI: 1.08–1.27)]. After adjusting by blood cell composition, b-Age remained significant in LAA etiology (Supplemental Table S3). For CE etiology (N = 242), the explanatory power of R-squared was 0.427 in model 1; neither b-Age nor c-Age were associated to mortality. In model 2, R-squared was 0.385; the most efficient model included c-Age [p = 0.001; OR = 1.07 (95% CI: 1.03–1.12)] and initial NIHSS [p < 0.001; OR = 1.19 (95% CI: 1.13–1.25)]. However, in model 3, b-Age and c-Age again lost statistical significance (Fig. 2, Supplemental Table S3). We could not replicate these stratified-by-subtype results because of the limited sample size in the replication cohort.

**Table 4:** Multivariate logistic regression models of mortality at 3 months after IS stratified by TOAST. Model 1 is the fully adjusted model, adjusted by clinical covariates and biological age. In model 2, stepwise logistic regression selected the most efficient model using the variables included in model 1.

		Model 1		Model 2	
		P- value	OR (95% CI) R^2^ = 0.373	P- value	OR (95% CI) R^2^_adj_ = 0.281
LAA (N = 153)	Biological age	0.004	1.14 (1.04–1.25)	0.006	1.10 (1.03–1.18)
	Chronological age	0.342	0.96 (0.88–1.05)	—	—
	Sex, female	0.754	0.79 (0.19–3.40)	—	—
	Recanalization treatment	0.208	0.32 (0.05–1.88)	—	—
	Previous mRS	0.363	1.26 (0.77–2.06)	—	—
	NIHSS	<0.001	1.22 (1.11–1.34)	<0.001	1.17 (1.08–1.27)
	Dyslipidemia	0.456	1.59 (0.47–5.34)	—	—
	Diabetes mellitus	0.417	1.62 (0.50–5.23)	—	—
	Atrial fibrillation	1.0	—	—	—
	Coronary heart disease	0.888	0.89 (0.17–4.74)	—	—
	Smoking habit	0.540	1.28 (0.58–2.86)	—	—
		**P- value**	**OR (95% CI) R** ^**2**^ ** = 0.427**	**P- value**	**OR (95% CI) R** ^**2**^ ** = 0.385**
CE (N = 242)	Biological age	0.261	1.04 (0.97–1.10)	—	—
	Chronological age	0.235	1.03 (0.98–1.10)	0.001	1.07 (1.03–1.12)
	Sex, female	0.822	0.90 (0.34–2.36)	—	—
	Recanalization treatment	0.583	1.28 (0.53–3.10)	—	—
	Previous mRS	0.060	1.28 (0.99–1.67)	—	—
	NIHSS	<0.001	1.18 (1.12–1.26)	<0.001	1.19 (1.13–1.25)
	Dyslipidemia	0.156	0.58 (0.27–1.23)	—	—
	Diabetes mellitus	0.785	1.11 (0.54–2.28)	—	—
	Atrial fibrillation	0.483	1.88 (0.32–10.8)	—	—
	Coronary heart disease	0.195	1.77 (0.75–4.21)	—	—
	Smoking habit	0.657	1.19 (0.56–2.54)	—	—

OR, odds ratio; CI, confidence interval; R^2^ adjusted-R squared (adjusted for the number of predictors in the model); NIHSS, National Institutes of Health Stroke Scale; mRS, modified Rankin Scale LAA, large-artery atherosclerosis; CE, cardioembolism.

## Discussion

We report that biological aging, an epigenetic biomarker estimated from DNAm values, better predicts mortality at 3 months after an IS event than chronological aging. This association is especially relevant in LAA stroke etiology. Our results are in line with previous studies in different diseases, which have shown that this biomarker predicts mortality independently of health status, lifestyle, and known genetic factors^13,15^.

Incidence of stroke is strongly correlated with increasing age^16^. In turn, age is a highly significant inverse predictor of functional outcome after IS, independent of stroke severity, characteristics, and complications^17^. Initial NIHSS, a well-validated tool for assessing initial stroke severity, has been postulated as the strongest predictor of mortality and functional outcomes after a stroke, followed by age; the traditional comorbidity index contributed little to the overall model^2–4^. Our results ratify these reports, as initial NIHSS was the main predictor of IS mortality, followed by b-Age or c-Age, depending on IS etiology and previous functional status.

The b-Age concept has been proposed as a better predictor of lifespan and functional capacity than c-Age alone^11,12,18^. In this context, b-Age seems more informative about the basal biological health of individuals. It could echo the burden of exposures (vascular risk factors, lifestyle habits, and environment) and provide an accurate measurement of the impact on the individual of these exposures that influence aging and potential longevity^18^.

Our results, replicated in an independent IS cohort, show that b-Age is an independent predictor of 3-month mortality, even after adjusting by c-Age and traditional vascular risk factors, baseline NIHSS, and blood cell composition. These results are in agreement with previous publications^13,15^ on epigenetic age acceleration (*AgeAccel)*, defined as the residual that results from regressing b-Age on c-Age. A positive *AgeAccel* value indicates that b-Age is higher than expected, based on c-Age^9,10^. In our study, we used the concept of b-Age because it is influenced by and informs both c-Age and *AgeAccel*.

However, the association between *AgeAccel* and mortality differs across IS etiology. Previous publications describe a favorable short-term prognosis after SVD, with low levels of 3-month mortality and reduced functional disability on hospital discharge, but also increased mid- and long-term risk of death, stroke recurrence, and dementia^19^. In LAA, initial NIHSS and b-Age are the main predictors of mortality. On the other hand, the role of b-Age as a predictor of mortality in CE stroke is not so clear. In one model, c-Age and initial NIHSS are the better predictors of mortality, but after adjusting by blood cell composition only initial NIHSS and previous mRS remain significantly associated. This may be due to c-Age and b-Age collinearity, as c-Age is highly correlated with blood cell types.

These differences between etiologies may be explained by the risk factors associated with each one. The prevalence of standard modifiable cardiovascular risk factors in IS differs by TOAST etiologies: patients with a CE stroke tend to be older than in other IS subtypes, and c-Age impact on outcome is more significant than the burden of vascular risk factors. On the other hand, patients with LAA etiology are younger and have more vascular risk factors (hypertension, diabetes mellitus, obesity, lipid disorders, and smoking) than other subtypes^16,20,21^. In a previous publication, we reported that the impact of b-Age is higher in younger people with stroke, possibly due to the accumulation of exposures (vascular risk factors, lifestyle habits, environment) that contribute to epigenetic changes with age, and because older individuals likely reach advanced ages because they have less biological damage^12^.

Early in life, DNA methylation can differ from the mean value because of genetic and environmental factors. However, blood DNA methylation decreases with increasing c-Age^6^. Epigenetic age varies due to clinical and lifestyle parameters: aging accelerates due to factors such as obesity, physical fitness, HIV infection, Parkinson disease and stroke^11,12,22–25^. Thus, one possible explanation for the association of advanced b-Age with increased IS mortality could lie in the accumulation of environmental exposures that may contribute to increased epigenetic changes with age, which accelerate aging. Biological age gathers these effects adding more information about the degree of damage in the organism than the mere chronological-age damage by itself. A more biologically aged individual would have less capacity to confront or to adapt to a serious injury like an acute stroke.

A major strength of the present study is that it evaluated the power of the associations of biological versus chronological age with 3-month IS mortality, and replicated those results in an independent cohort. Some limitations of this novel study should be considered. We measured methylation levels in peripheral blood-cell DNA; for some CpGs, the methylation is tissue-specific^26^. Therefore, we could have lost signals by not choosing tissues where epigenetic age may have greater repercussions on stroke outcome and mortality at 3 months, which would be the ideal situation. However, methylation patterning of whole blood has been described as a good approximation to the target tissue that is to be studied, since it has shown a good correlation with the methylation pattern of other tissues^6,27,28^. In the case of atherosclerosis, previous epigenetic studies carried out in peripheral blood samples and in aorta tissue showed a consistent direction of their associations in epigenome wide association studies (EWAS)^29–32^. In addition, b-Age predictor used is based on whole blood DNA, being the most efficient tissue to estimate b-Age^9^. In this cross-sectional study, we cannot establish the causality of biological aging, but we can use these findings as potential biomarkers of IS mortality. Finally, the association of biological aging in LAA with mortality must still be replicated.

In conclusion, biological age better predicts IS mortality at 3 months than chronological age, particularly in LAA stroke etiology. Epigenetic age could be considered a useful biomarker to predict the risk of mortality after a stroke.

## Materials and Methods

### Study Participants

The study included a prospective cohort of Caucasian IS patients from Hospital del Mar in Barcelona, Spain, analyzed retrospectively, recruited from 2009 to 2013 (BASICMAR)^33,34^. The replication cohort was provided by Vall d’Hebron (VH). Inclusion criteria for both cohorts were as follows: (1) brain imaging with computed tomography(CT) and magnetic resonance imaging (MRI) in the acute phase, (2) clinical data supporting the assigned stroke subtype according to TOAST classification^35^ and (3) absence of intracranial hemorrhage, neoplasms, demyelinating and autoimmune diseases, and vasculitis. All patients were assessed and classified by a neurologist and were included in the study by consecutive order of recruitment.

The study was approved by the ethics committees (CEIC) of Parc de Salut Mar and Vall d’Hebron Hospital, Barcelona. All participants (or their approved proxy) provided written informed consent for participation. The study was conducted according to the principles expressed in the Declaration of Helsinki and relevant national legislation.

### Clinical Variables

All patients were evaluated at hospital admission by a neurologist and received a CT or MRI scan in the emergency room. Functional independence previous to IS was recorded as p-mRS score. In the VH replication cohort, functional independence was classified as a categorical variable, independent (p-mRS ≤ 2) or dependent (p-mRS > 2); all patients in the replication cohort were functionally independent previous to IS. Initial severity was evaluated using NIHSS. Recanalization involved tissue plasminogen activator (r-tPA), first 4.5 h, or endovascular treatment. TOAST classification was recorded^35^.

Data on vascular risk factors were obtained from direct interview of the patient, relatives, and caregivers, from medical records, and were recorded as defined in international guidelines, as previously described^34^.

Mortality data were obtained from electronic medical records, hospital admissions records, or by telephone contact with primary care physicians or family members.

### Array-based DNA Methylation Analysis with Infinium Human Methylation 450 k

DNA samples were extracted from whole peripheral blood collected in 10 mL EDTA tubes at hospital arrival, in the acute phase of the stroke (maximum within 12 hours of symptoms onset). Genome-wide DNAm was assessed using the Illumina Human Methylation 450 Beadchip (Illumina Netherlands, Eindhoven, Netherlands), following the manufacturer protocol. All these processes were done at Progenika Biopharma (Bizkaia, Spain).

Data were pre-processed using standardized pipelines^36,37^. Sample and CpG quality controls and the statistical analysis were performed as described in Soriano-Tarraga *et al*.^34^. We used a previously published Houseman algorithm to infer white blood cell counts from DNAm data^10,38^.

### Biological Age and Epigenetic Clock

Biological age was calculated using the DNAm levels of whole-blood DNA. We chose the Hannum method because it better estimated age from whole blood, as we previously reported^12^. This method is based on 71 methylation probes from the Illumina 450 K Methylation array, derived as the best predictors of b-Age using data generated from whole blood^9^. The sum of the beta values multiplied by the reported effect sizes for the Hannum predictor yielded b-Age.

The concept of age acceleration (AgeAccel) is defined as the residuals from the linear regression of DNAm age on chronological age in control samples^15^. This variable, AgeAccel, was not correlated with c-Age and takes on a positive value for samples whose DNAm age is higher than expected. Additionally, we adjusted AgeAccel by sex, p-mRs, NIHSS and TOAST (defined as the residuals from the linear regression of DNAm age on c-Age in control samples, sex, p-mRs, NIHSS, and TOAST) to illustrate graphically the results of the statistic models.

### Statistical Analysis

Continuous variables are presented as means and standard deviation (SD) or medians and interquartile ranges (IQR), and categorical variables as absolute values and percentages. For the bivariate analyses, baseline characteristics of IS and 3-month mortality were compared using Student t-test for continuous variables and χ2 test for categorical variables.

Three-month mortality was analyzed by multivariate logistic regression. Three statistical models were used in the discovery cohort. Model 1, the full model, was adjusted for the characteristics that were most representative and statistically associated (p ≤ 0.05) with mortality. Model 2 was the most efficient model, selected by stepwise regression. Model 3 (sensitivity analysis) was further adjusted by blood cell composition^10,38^. Using these models, we also stratified by TOAST classification. Calibration of the multivariate logistic models was assessed with the Hosmer–Lemeshow test. A high p-value indicates a good fit for the model.

All statistical analysis was performed using R statistical package, version 3.2^39^, STATA, and SPSS version 18.0. All analysis was two-tailed. Statistical significance was set at a p-value of 0.05.

### Data availability

The dataset analysed during the current study are available in the Gene Expression Omnibus (GEO, http://www.ncbi.nlm.nih.gov/geo/) under accession number GSE69138.

### Accession number

The dataset are available in the Gene Expression Omnibus under accession number GSE69138.

## Electronic supplementary material

Supplementary Tables
