# Supplementary material for: Biological Age is a predictor of mortality in Ischemic Stroke
Source: Sci Rep. 2018 Mar 7;8:4148. doi: 10.1038/s41598-018-22579-0 (PMC5841388; doi:10.1038/s41598-018-22579-0)
Supplement: Supplementary file 1 — Supplementary Tables [file 41598_2018_22579_MOESM1_ESM.pdf]

ONLINE SUPPLEMENT

Biological Age is a predictor of mortality in Ischemic Stroke.

Authors:

Carolina Soriano-Tárraga PhD[1], Eva Giralt-Steinhauer MD PhD[1], Marina Mola-Caminal PhD[1], Angel Ois MD PhD[1], Ana Rodríguez-Campello MD[1], Elisa Cuadrado-Godia MD PhD[1], Israel Fernández-Cadenas PhD[2], Natalia Cullell MSc[2], Jaume Roquer MD PhD[1*] and Jordi Jiménez-Conde MD PhD[1*]

1.-Department of Neurology, Hospital del Mar; Neurovascular Research Group, IMIM (Institut Hospital del Mar d'Investigacions Mèdiques); Universitat Autònoma de Barcelona/DCEXS-Universitat Pompeu Fabra, Barcelona, Spain

2.- Stroke Pharmacogenomics and Genetics, Fundació Docència i Recerca, MutuaTerrassa, Hospital Mútua de Terrassa, Terrassa, Spain

Supplemental Table S1. **Mortality at 3 months after ischemic stroke (Replication cohort).** Bivariate analysis. NIHSS, National Institutes of Health Stroke Scale.

	**Alive (N=70)**	**Deceased (N=15)**	**p-value**
**Chronological age (years)***	72 (65-78)	80 (72-86)	0.007
**Biological age (years)***	79.9 (73.8-84.9)	89.2 (81.0-94.1)	0.002
**Sex (female), n (%)**	18 (25.7)	7 (46.7)	0.106
**NIHSS***	6.5 (2-14)	17 (14-21)	<0.001
**Recanalization treatment, n (%)**	29 (43.3)	13 (86.7)	0.002
**Dyslipidemia, n (%)**	31 (44.9)	4 (28.6)	0.259
**Hypertension, n (%)**	42 (60.0)	11 (73.3)	0.333
**Diabetes mellitus, n (%)**	17 (24.3)	3 (20.0)	0.723
**Coronary heart disease, n (%)**	9 (13.0)	6 (40.0)	0.013
**Atrial fibrillation, n (%)**	10 (14.7)	10 (66.7)	<0.001
**Smoking habit, n (%)**	18 (26.1)	2 (14.3)	0.347
**Ischemic stroke etiology, n (%)**			0.001
**Large-artery atherosclerosis**	30 (43.5)	5 (33.3)	
**Small-vessel disease**	8 (11.6)	0 (0)	
**Cardioembolism**	10 (14.5)	9 (60.0)	
**Undetermined**	21 (30.4)	1 (6.7)	

NIHSS, National Institutes of Health Stroke Scale

Supplemental Table S2. **Ischemic stroke etiology (TOAST).** Bivariate analysis stratified by TOAST.

	LAA (N=153)	SVD (N=199)	CE (N=242)	p-value
**Chronological age (years)***	75 (64-80)	73 (64-80)	80 (73-85)	<0.001
**Sex (female), n (%)**	51 (33.3)	67 (33.5)	150 (62.0)	<0.001
**Previous mRS***	0 (0-1)	0 (0-1)	0 (0-2)	<0.001
**NIHSS***	5 (3-10)	3 (2-5)	9 (5-18)	<0.001
**Mortality n (%)**	19 (12.4)	1 (1.1)	74 (30.6)	<0.001
**Recanalization treatment, n (%)**	26 (17.0)	17 (8.5)	54 (22.3)	<0.001
**Dyslipidemia, n (%)**	85 (55.6)	97 (48.5)	94 (38.8)	0.004
**Hypertension, n (%)**	106 (69.3)	142 (71.0)	182 (75.2)	0.390
**Diabetes mellitus, n (%)**	77 (50.3)	79 (39.5)	92 (38.0)	0.040
**Coronary heart disease, n (%)**	23 (15.0)	21 (10.5)	46 (19.2)	0.041
**Atrial fibrillation, n (%)**	2 (1.3)	1 (0.5)	217 (89.7)	<0.001
**Smoking habit, n (%)**	56 (36.6)	52 (26.0)	30 (12.5)	<0.001

*Median (Interquartile range)
† Mean (Standard deviation)
TOAST, Trial of Org 10172 in Acute Stroke Treatment; NIHSS, National Institutes of Health Stroke Scale; mRS, modified Rankin Scale; LAA, large-artery atherosclerosis; SVD, small-vessel disease, CE, cardioembolism.

Supplemental Table S3. Logistic multivariate regression of 3-month mortality, stratified by ischemic stroke etiology. Fully adjusted model with results for all blood cell compositions.

	LAA (N=153)		CE (N=238)	
	P-value	OR (CI 95%)	P-value	OR (CI 95%)
**Biological age**	0.033	1.14 (1.01-1.28)	0.574	-
**Chronological age**	0.190	-	0.362	-
**Sex, female**	0.788	-	0.992	-
**Recanalization treatment**	0.184	-	0.596	-
**Previous mRS**	0.711	-	0.039	1.36 (1.02-1.82)
**NIHSS**	0.001	1.27 (1.10-1.46)	<0.001	1.21 (1.13-1.29)
**Dyslipidemia**	0.347	-	0.227	-
**Diabetes mellitus**	0.537	-	0.606	-
**Atrial fibrillation**	0.999	-	0.675	-
**Coronary heart disease**	0.654	-	0.395	-
**Smoking habit**	0.891	-	0.606	-
**Monocytes**	0.047	0.64 (0.41-0.99)	0.113	-
**NK**	0.258	-	0.856	-
**B cells**	0.812	-	0.650	-
**Granulocytes**	0.513	-	0.301	-
**CD4 T cells**	0.299	-	0.247	-
**CD8 T cells**	0.432	-	0.319	-
**naïve CD8 T cells**	0.039	0.97 (0.94-1.00)	0.764	-
**naïve CD4 T cells**	0.885	-	0.214	-
**CD8+CD28-CD45RA-**	0.768	-	0.497	-
**Plasmablast**	0.551	-	0.021	36.2 (1.73-758)

NIHSS, National Institutes of Health Stroke Scale; NK, natural killer cell; OR, odds ratio; CI, confidence interval; mRS, modified Rankin Score; OR, odds ratio; LAA, large-artery atherosclerosis; CE, cardioembolism; SVD, small-vessel disease.
